# Loneliness among older adults with chronic diseases and multimorbidity: a scoping review of prevalence and associated factors

**DOI:** 10.3389/fpubh.2026.1846531

**Published:** 2026-07-31

**Authors:** Menglu Wang, Yu Ni, Man Xiang, Fangfang Peng, Xuemei Gu, Lihui Huang, Shiyao Yang, Yue Qi, Xiuchuan Li

**Affiliations:** 1 School of Nursing, Chengdu Medical College, Chengdu, China; 2 Department of Cardiology, Chinese People’s Liberation Army Western Theater General Hospital, Chengdu, China; 3 School of Nursing, Chengdu University of Traditional Chinese Medicine, Chengdu, China

**Keywords:** associated factors, chronic diseases, loneliness, multimorbidity, older adults, scoping review

## Abstract

**Background:**

Loneliness is increasingly recognized as an important psychosocial health issue among older adults. Older adults with chronic diseases and multimorbidity may be particularly vulnerable because long-term disease burden, coexisting chronic conditions, functional limitations, and reduced social participation often interact. This scoping review aimed to map the available evidence on assessment tools, prevalence estimates, and factors associated with loneliness among older adults with chronic diseases and multimorbidity.

**Methods:**

A systematic search was conducted in CNKI, Wanfang, VIP, China Biomedical Literature Database, Embase, The Cochrane Library, PubMed, Web of Science, and CINAHL from database inception to November 2025. Eligible studies were screened and extracted according to predefined criteria. Descriptive statistics, narrative synthesis, and thematic analysis were used to summarize the findings.

**Results:**

Twenty-one studies involving 26,140 participants were included. Five loneliness assessment approaches were identified, including the UCLA Loneliness Scale, the 3-item UCLA Loneliness Scale, the ULS-8 Scale, the De Jong Gierveld Loneliness Scale, and a single-item loneliness question. Thirteen studies reported prevalence estimates, ranging from 25.0 to 97%. When stratified by assessment instrument, estimates were generally higher in studies using the full UCLA Loneliness Scale and lower in studies using single-item loneliness questions. Factors associated with loneliness were grouped into four categories: sociodemographic factors, health status and disease-related factors, lifestyle and social support factors, and psychological and cognitive factors. Insufficient social support, chronic disease burden, multimorbidity, functional impairment, depression, anxiety, living alone, and widowhood were repeatedly reported.

**Conclusion:**

Loneliness among older adults with chronic diseases and multimorbidity is common but heterogeneous across studies. Future research should standardize measurement approaches and conduct longitudinal and intervention studies to inform integrated and personalized management strategies.

**Systematic review registration:**

Identifier md2je. The review was registered with the Open Science Framework. The publicly accessible registration URL is: https://osf.io/md2je/.

## 1 Background

With the acceleration of global population aging, the proportion of people aged 60 years and above continues to increase. Health issues in later life have become increasingly prominent, and the mental health of older adults also warrants greater attention. According to the United Nations *World Population Prospects 2024* (1), population aging is increasingly characterized by advanced age, the risk of disability among older adults has increased substantially, and average life expectancy has risen to 79.0 years. More than 60% of older adults have at least one chronic disease, and 25.2% have three or more coexisting diseases. Chronic diseases and multimorbidity may have long-term, cumulative, and potentially irreversible impacts on older adults and may be associated with increased risks of cardiovascular disease, dementia, and even suicidal tendency (2). These findings indicate that the world is facing the dual pressures of population aging and the high burden of chronic diseases and multimorbidity, resulting in a rapidly increasing need for mental health management among older adults. Loneliness is a negative emotional experience that arises when an individual’s desired social relationships and emotional needs are not adequately met. Commonly used tools for assessing loneliness include the University of California Los Angeles Loneliness Scale (UCLA Loneliness Scale), the 3-item short-form UCLA Loneliness Scale, and the De Jong Gierveld Loneliness Scale. Among these, the UCLA Loneliness Scale is the most widely used instrument. It has good reliability and validity, covers both emotional and social dimensions, and is considered a standard tool in clinical and research settings. It is applicable to adults, older adults, college students, and the general population. The 3-item short-form UCLA Loneliness Scale is brief and is commonly used in large-scale surveys, such as community-based investigations, making it suitable for rapid screening among adults and older adults. The De Jong Gierveld Loneliness Scale is available in 3-item and 6-item versions and distinguishes between emotional loneliness and social loneliness. It is suitable for exploring the mechanisms of loneliness among older adults and general adult populations. Although these instruments have deepened our understanding of loneliness; they differ in target populations, assessment dimensions, and applicable settings. An increasing number of studies have explored the association between chronic diseases and loneliness. However, owing to differences in study design, assessment instruments, geographical regions, and population characteristics, consensus has not yet been reached regarding the prevalence, detection rate, or reported proportion of loneliness and its associated factors among older adults with chronic diseases and multimorbidity. Previous studies have shown (3) that loneliness is common among older adults and represents a persistent psychological issue for older adults with chronic diseases and multimorbidity. It may be associated with increased long-term care needs, higher readmission risk, delayed recovery, poorer long-term health outcomes, and increased mortality risk. Among older adults with chronic diseases and multimorbidity, disease burden, functional limitations, repeated healthcare visits, and reduced social participation may interact with each other, making this population more vulnerable to persistent loneliness. Although the adverse consequences of loneliness among older adults with chronic diseases and multimorbidity have been widely recognized, existing domestic and international evidence remains highly heterogeneous in terms of research methods, assessment tools, population characteristics, and cultural contexts. As a result, estimates of loneliness prevalence vary substantially across studies, and findings regarding associated factors remain inconsistent. Therefore, following the scoping review framework proposed by Arksey and O’Malley (4), this review systematically maps the assessment tools, prevalence, detection rate or reported proportion, and associated factors of loneliness in this population. The findings are expected to provide evidence for loneliness risk identification, population stratification, and the design of future nursing intervention studies in clinical practice.

## 2 Methods

The methods of this scoping review followed the framework outlined by Arksey and O’Malley (4) and further developed by Levac et al. (5), which includes five stages: identifying the research question, identifying relevant studies, selecting studies, charting the data, and collating, summarizing, and reporting the results. This review aimed to describe loneliness among older adults with chronic diseases and multimorbidity and to report factors associated with loneliness in this population. This review was conducted in accordance with the Joanna Briggs Institute (JBI) methodology and reported according to the PRISMA Extension for Scoping Reviews (PRISMA-ScR) checklist. A basic methodological quality appraisal of the included studies was conducted using the appropriate JBI critical appraisal tools according to study design. Analytical cross-sectional studies were assessed using the JBI Critical Appraisal Checklist for Analytical Cross-Sectional Studies (6), prospective cohort studies using the JBI Critical Appraisal Checklist for Cohort Studies (7), and case–control studies using the JBI Critical Appraisal Checklist for Case–Control Studies (8). Two reviewers independently appraised each study. Each item was rated as “yes,” “no,” “unclear,” or “not applicable,” and disagreements were resolved through discussion or consultation with a third reviewer. The appraisal results were not used as criteria for excluding studies but were used to inform the interpretation of the credibility and limitations of the evidence. The methodological quality appraisal of the included studies is presented in Supplementary Table S1.

The review protocol was registered with the Open Science Framework (OSF; registration number: md2je).

### 2.1 Aim and review questions

After reviewing the literature, the following research questions were formulated: (a) What assessment tools are currently used to measure loneliness? (b) What is the prevalence, detection rate, or reported proportion of loneliness among older adults with chronic diseases and multimorbidity in China and other countries? (c) What factors are associated with loneliness among older adults with chronic diseases and multimorbidity?

### 2.2 Search strategy

A combination of subject headings and free-text terms was used to search the following databases: China National Knowledge Infrastructure (CNKI), Wanfang Database, VIP Database, China Biomedical Literature Database (CBM), Embase, The Cochrane Library, PubMed, Web of Science, and CINAHL. The search period covered all records from database inception to November 2025. Search terms included “Aging/Old*/Elder*/Geriatric*/Aged patient/Older adults,” “Chronic disease/Chronic illness*/Chronic condition*/Noncommunicable disease,” and “Loneliness/Lonely/Homesickness/Emotional loneliness/Social loneliness.” The complete search strategies for each database are provided in Supplementary Table S2.

### 2.3 Eligibility criteria

#### 2.3.1 Inclusion criteria

(a) The study design was observational, including cross-sectional studies, cohort studies, or case–control studies. (b) Participants were older adults aged 60 years and above who had at least one chronic disease or multimorbidity, as confirmed by clinical diagnosis or study records. Chronic diseases are defined as non-communicable conditions with a long disease course that usually require ongoing management, such as cardiovascular disease, stroke, diabetes, chronic respiratory disease, malignant tumors, chronic kidney disease, and hypertension. Multimorbidity was defined as the coexistence of two or more chronic diseases in the same individual. (c) The outcomes included the prevalence, detection rate, or reported proportion of loneliness and/or factors associated with loneliness. (d) The study used at least one loneliness assessment tool for screening or measurement. (e) The publication was written in Chinese or English.

#### 2.3.2 Exclusion criteria

(a) Reviews, grey literature, editorials, non-peer-reviewed journal content, letters to the editor, and study protocols were excluded to ensure peer review, verifiability of sources, completeness of methodological information, and consistency of data extraction. (b) Studies with data that were difficult to extract or contained obvious errors were excluded. (c) Studies that did not report the corresponding outcomes were excluded.

### 2.4 Data extraction and analysis

All retrieved records were imported into EndNote X9 and duplicates were removed through both automated and manual procedures. Two reviewers independently screened the titles and abstracts according to the inclusion and exclusion criteria. After the initial screening, full texts were obtained for further assessment. Any disagreements that could not be resolved by consensus were settled through discussion or consultation with a third reviewer. Data were then extracted, including author, publication year, country, study design, sample source, sample size, survey period, and participant age. Age information was extracted as reported in the original studies, including mean ± standard deviation, median with interquartile range or range, and age eligibility thresholds. Data not reported in the original articles were uniformly marked as NR. The loneliness assessment tool used, the prevalence, detection rate, or reported proportion of loneliness, and factors associated with loneliness were also extracted. After data extraction, the reviewers jointly developed a data-charting form in Excel tailored to the research questions. Throughout the review process, descriptive statistics and narrative synthesis were used to organize and synthesize the findings (9), and thematic analysis was applied to classify factors associated with loneliness (10).

## 3 Results

### 3.1 Search outcome

**Figure 1:**
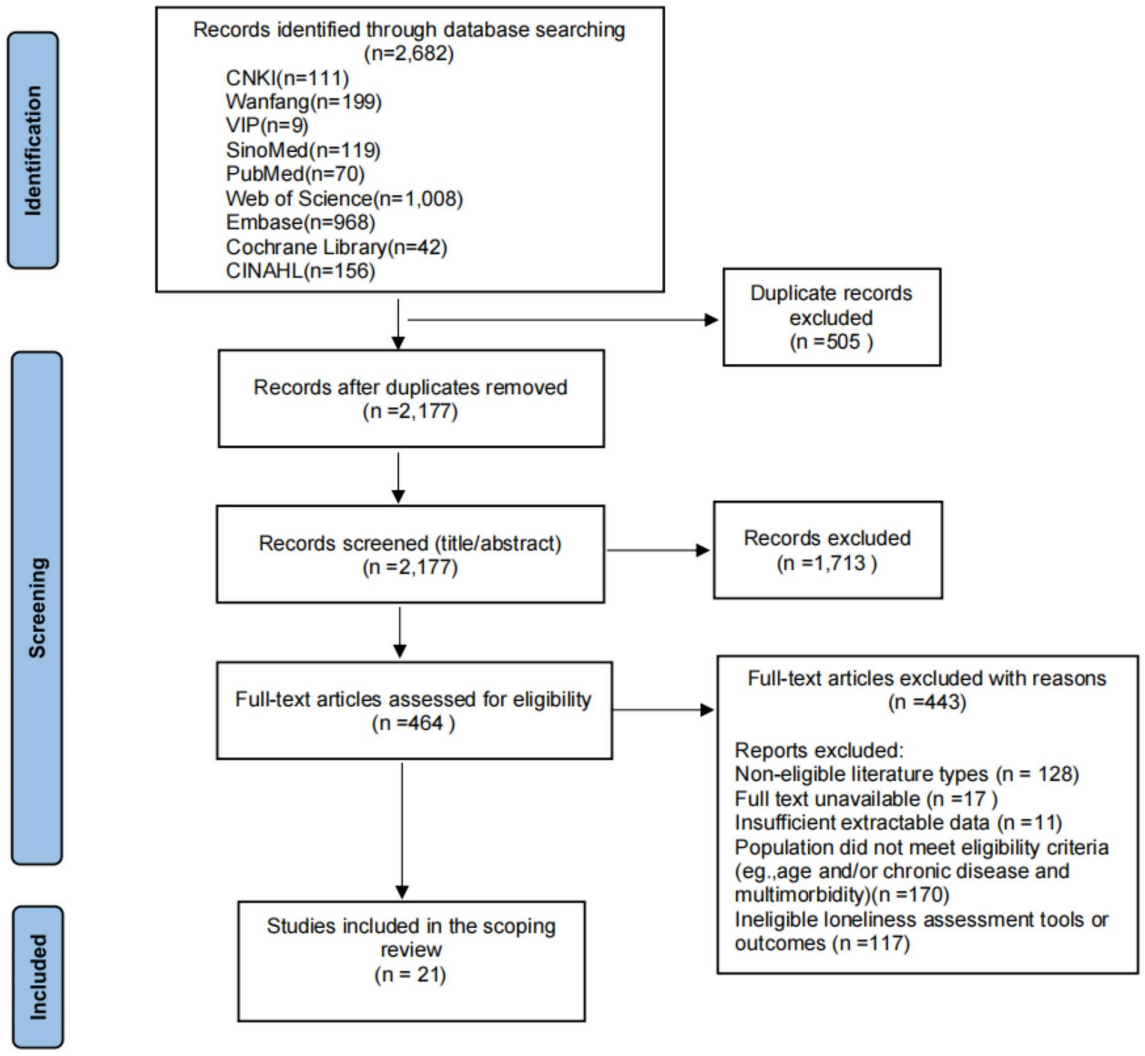
PRISMA flowchart.

The initial search identified 2,682 records. After removing 505 duplicates, 1,713 records were excluded by screening titles and abstracts. A further 443 articles were excluded after full-text review. Finally, 21 studies were included (11–31), with a total sample size of 26,140 participants. The study selection process is shown in Figure 1.

### 3.2 Description of studies

A total of 21 studies were included, comprising 10 Chinese-language studies and 11 English-language studies, all published between 2007 and 2025. The included studies were mainly conducted in China (*n* = 13), with the remaining studies conducted in Türkiye (*n* = 2), the United States (*n* = 2), Italy (*n* = 1), Japan (*n* = 1), the United Kingdom (*n* = 1), and India (*n* = 1). Most studies were cross-sectional studies (*n* = 18), while the remaining studies included prospective cohort studies (*n* = 2) and a case–control study (*n* = 1). The study settings were mainly hospitals (*n* = 10), followed by community settings (*n* = 6), home-based settings (*n* = 2), nursing homes or long-term care institutions (*n* = 1), and rural areas (*n* = 1); one study did not report the study setting. Twelve studies used the UCLA Loneliness Scale, three used the 11-item De Jong Gierveld Loneliness Scale, three used the 3-item short-form UCLA Loneliness Scale, two used a single question, “Do you feel lonely?,” and one used the ULS-8 scale. All studies included participants with at least one chronic non-communicable disease. The basic characteristics of the included studies are presented in Table 1.

**Table 1:** Basic characteristics of the included studies.

Author and year and publication language	Country/region	Study design	Sample source	Sample participants	Age, years	Survey period	Tool	Associated factors	Prevalence of loneliness
Huang Hairong et al., 2017 (11) Chinese	China	Cross-sectional study	Nursing homes / long-term care institutions	597	Female: 72.4 ± 7.5; Male: 74.3 ± 8.0	2015–2016	UCLA	Male sex, age, chronic disease, living alone, lack of social activities, and education level	87.60%
Zou Jiayu et al., 2021 (12) Chinese	China	Cross-sectional study	Home setting	3,250	≥65 years	NR	UCLA	Community satisfaction, childlessness, education level, rural residence, marital status, pre-retirement occupation, and alcohol abstinence	66.90%
Li Fan et al., 2007 (13) Chinese	China	Cross-sectional study	Hospital	60	76.42 ± 6.45	NR	UCLA	Length of hospital stay, monthly household income, education level, activities of daily living (ADL), self-rated health score, interpersonal relationships, and marital status	96.70%
Zhang Yuting et al., 2024 (14) Chinese	China	Cross-sectional study	Community	736	≥60 years	2022.07–2022.09	UCLA	Age, education level, marital status, monthly household income, living arrangement, social support, death anxiety, and pre-retirement occupation	Moderate level
Yang Huanhuan et al., 2022 (15) Chinese	China	Descriptive study	Hospital	320	71.05 ± 8.66	2021.05–2021.10	UCLA	Farmer occupation, marital status, type of medical insurance, disease status, having ≥2 diseases, and spouse as the main source of income	80.30%
Cheng Shuai et al., 2022 (16) Chinese	China	Cross-sectional study	Hospital	325	61.66 ± 12.16	2021.05–2021.10	UCLA	Age, education level, place of residence, comorbid chronic diseases, daily life management, rehabilitation exercise management, and social support	84.00%
Huai Yijie et al., 2025 (17) Chinese	China	Cross-sectional study	Hospital	175	69.98 ± 10.27	2022.06–2023.02	ULS-8	Anxiety level, utilization of social support, number of caregivers, and subjective support	52.60%
Li Chao et al., 2018 (18) Chinese	China	Cross-sectional study	Community	1,550	> 60 years	NR	UCLA	Age, marital status, living arrangement, annual income, education level, relationship with neighbors, help-seeking methods, communication methods, and participation in group activities	Moderate level
Qin Yuqian et al., 2023 (19) Chinese	China	Cross-sectional study	Community	482	≥60 years	2021.12–2022.10	UCLA	Sex, living arrangement, hearing status, sleep status, family function, and social support factors	Moderate level
Liu Xi et al., 2022 (20) Chinese	China	Cross-sectional study	NR	7,212	82.9 ± 10.91	2018	Single-item question	Self-rated health score, limitations in daily activities, having ≥3 diseases, education level, family function, marital status, income, neighborhood relationships, physical exercise, and community support	26.30%
Vespa et al., 2023 (21) English	Italy	Prospective cohort study	Hospital	216	71.6 ± 5.5	NR	11-item De Jong Gierveld Loneliness Scale	Social support, emotional health, functional health, age, having ≥3 diseases, and social relationships	NR
SYS Wong et al., 2020 (22) English	China	Prospective cohort study	Hospital	583	70.9 ± 6.1	2016.6–2020.4	11-item De Jong Gierveld Loneliness Scale	Female sex, living alone, and number of chronic diseases	70.10%
Jiao Zhang et al., 2018 (23) English	China	Cross-sectional study	Rural area	5,514	69.7 ± 6.5	2017	Single-item question	Age, marital status, education level, living arrangement, income, self-rated health status, number of chronic diseases, and activities of daily living (ADL)	25.00%
Sandeep Grover et al., 2019 (24) English	North India	Cross-sectional study	Hospital	296	≥60 years	NR	UCLA	Social network support, personality, psychological factors, living alone, age, disease status, generalized anxiety disorder, and abuse	55.40%
Emma Cho et al., 2024 (25) English	United States	Cross-sectional study	Community	541	67 ± 9.8	2015–2016	3-item UCLA Loneliness Scale	Female sex, income, marital status, and depression	38%
Theeke et al., 2013 (26) English	United States	Cross-sectional study	Community	60	75.2 ± 6.42	2009	UCLA	Types of chronic diseases, including mood disorders, pulmonary disease, and heart disease; number of diseases; benzodiazepine use; income; education level; living arrangement; and quality of life	97.00%
Hacihasanoğlu et al., 2012 (27) English	Turkey	Cross-sectional study	Hospital	830	≥65 years	2010.3–2010.6	UCLA	Marital status, education level, income, living alone, number of chronic diseases, health status, family function, living arrangement, and interpersonal relationships	High level
Chunmei Xia et al., 2023 (28) English	China	Case–control study	Hospital	324	63.0 ± 9.8	2021.3–2022.7	UCLA	Marital status, disease status, and systemic treatment	Moderate level
Ohta et al., 2024 (29) English	Japan	Cross-sectional study	Hospital	647	71.26 ± 12.18	2023.9–2023.11	3-item UCLA Loneliness Scale	Social support participation and body mass index (BMI)	52.10%
Sönmez Sari et al., 2024 (30) English	Turkey	Cross-sectional study	Home setting	370	75.95 ± 8.49	NR	11-item De Jong Gierveld Loneliness Scale	Polypharmacy, female sex, education level, marital status, income, rural residence, living alone, family relationships, and number of chronic diseases	NR
Kandola et al., 2023 (31) English	United Kingdom	Prospective cohort study	Community	2052	63 ± 10	NR	3-item UCLA Loneliness Scale	Age, sex, marital status, education level, economic status, occupational status, BMI, physical activity, smoking, drinking history, cognitive function, and depression	Low level

“NR” indicates that the information was not reported or could not be extracted from the original article. UCLA Loneliness Scale: University of California, Los Angeles Loneliness Scale.11-item De Jong Gierveld Loneliness Scale: De Jong Gierveld Loneliness Scale, 11-item version.3-item UCLA Loneliness Scale: Three-item short form of the University of California, Los Angeles Loneliness Scale. ULS-8 Scale: Eight-item short form of the University of California, Los Angeles Loneliness Scale. Single-item question: “Do you feel lonely?”.

Age data are presented as reported in the original studies. Values are shown as mean ± standard deviation, median with interquartile range or range, or age eligibility criteria when only an age threshold was reported.

### 3.3 Loneliness among older adults with chronic diseases and multimorbidity

Thirteen studies reported the prevalence, detection rate, or reported proportion of loneliness among older adults with chronic diseases and multimorbidity, ranging from 25.0 to 97.0%. Owing to differences in assessment tools, diagnostic or cut-off criteria, study settings, and sample characteristics across the included studies, this range should not be interpreted as directly pooled epidemiological estimates, but rather as descriptive findings reflecting the burden of loneliness in different research contexts. When stratified by assessment tool, studies using the UCLA Loneliness Scale reported loneliness proportions ranging from 55.4 to 97.0%; the study using the ULS-8 scale reported a proportion of 52.6%; studies using the 3-item short-form UCLA Loneliness Scale reported proportions ranging from 38.0 to 52.1%; the study using the De Jong Gierveld Loneliness Scale reported a proportion of 70.1%; and studies using the single question “Do you feel lonely?” reported proportions ranging from 25.0 to 26.3%. Overall, multi-item scales, particularly the full UCLA Loneliness Scale, tended to yield relatively higher reported proportions of loneliness, whereas single-item questions and short-form scales tended to yield relatively lower estimates. This suggests that measurement instruments and cut-off criteria may be important sources of variation in reported loneliness proportions. When stratified by study setting, studies conducted in hospitals or disease-specific clinical settings generally involved older patients with heavier disease burden, functional limitations, or greater treatment needs, whereas studies conducted in community, home-based, or rural settings more often reflected the loneliness experiences of older adults with chronic diseases in their daily living environments. Because study settings were also influenced by differences in disease type, functional status, level of social support, and measurement tools, this review did not directly pool loneliness proportions across settings. Instead, the findings were presented using a stratified narrative approach.

### 3.4 Factors associated with loneliness among older adults with chronic diseases and multimorbidity

Among the 21 included studies, multiple studies reported factors associated with loneliness among older adults with chronic diseases and multimorbidity from different perspectives. After data extraction and synthesis, these factors were categorized into four domains: social-demographic factors, health status and disease-related factors, lifestyle and social support factors, and psychological and cognitive factors, involving 16 distinct variables. The results are presented in Table 2.

**Table 2:** Associated factors of loneliness among older adults with chronic diseases and multimorbidity.

Theme	Specific factors
Social-demographic factors	Gender, Age, Marriage Status, Educational Attainment, Economic Status, Residence and Type of Medical Insurance
Health status and disease-related factors	Chronic Disease Characteristics, Symptoms and Functional impairment, Self-rated Health, Treatment-related Factors
Lifestyle and social support factors	Social Support, Family Function and Interpersonal Relationships, Social Participation and Activity Level, Community and Environmental Resources
Psychological and cognitive factors	Psychological Symptoms, Cognitive Level

#### 3.4.1 Social-demographic factors

(1) Marital status. Eleven studies (12–15, 18, 23, 25, 27, 28, 30, 31) reported that being divorced or single was an important factor associated with loneliness. (2) Educational level. Ten studies (11–14, 16, 18, 20, 26, 30, 31) reported that higher educational level was associated with a lower prevalence, detection rate, or reported proportion of loneliness. (3) Economic income. Eleven studies (13–15, 18, 20, 23, 25–27, 30, 31) reported that lower income was associated with a higher prevalence, detection rate, or reported proportion of loneliness. (4) Age. Seven studies (12, 15, 17, 19, 24, 25, 31) reported age as an important factor associated with loneliness, with older age being associated with a higher prevalence, detection rate, or reported proportion of loneliness. (5) Sex. Six studies (11, 19, 22, 23, 30, 31) reported an association between sex and loneliness; however, the direction of this association was not fully consistent across studies. (6) Other factors. Other indicators included place of residence, occupational type, and socioeconomic status.

#### 3.4.2 Health status and disease-related factors

(1) Number of coexisting chronic diseases. Ten studies (11, 15, 16, 20–23, 26, 27, 30) reported that a greater number of coexisting chronic diseases may indicate a higher likelihood of loneliness. (2) Chronic disease symptoms. Five studies (15, 20, 24, 26, 28) reported that disease duration, symptom severity, and symptom control were associated with higher levels of loneliness. (3) Activities of daily living/functional status. Six studies (13, 16, 20, 21, 23, 31) reported that lower levels of activities of daily living or poorer functional status were associated with loneliness, with poorer daily functioning being related to a higher prevalence, detection rate, or reported proportion of loneliness. (4) Self-rated health. Five studies (13, 20, 21, 23, 27) reported self-rated health as an important factor associated with loneliness. (5) Medication use. Two studies (26, 30) reported that medication use was closely associated with loneliness. (6) Other factors. Hearing status and sleep status were also reported as factors associated with higher levels of loneliness.

#### 3.4.3 Lifestyle and social support factors

(1) Level of social support. Eight studies (12, 16, 17, 19–21, 24, 29) reported that social support was an important independent factor associated with loneliness. (2) Family function and interpersonal relationships. Six studies (13, 18–20, 28, 30) reported that support from family members, spouses, or children, frequency of contact with relatives and friends, and interaction with others were associated with a lower prevalence, detection rate, or reported proportion of loneliness. (3) Living arrangements and environmental factors. Nine studies (11, 14, 18–20, 22, 23, 27, 30) reported that older adults with chronic diseases and multimorbidity who lived alone, lived in empty-nest households, or lived in areas with relatively limited social resources generally had higher levels of loneliness. (4) Level of social participation. Two studies (11, 18) reported that participation in cultural, recreational, religious, or community activities, as well as the degree of engagement in social networks, was closely associated with loneliness. (5) Quality of life. One study (26) reported quality of life as an important factor associated with loneliness.

#### 3.4.4 Psychological and cognitive factors

(1) Negative emotions. Five studies (17, 21, 24, 25, 31) reported that emotions such as anxiety and depression often co-occurred with loneliness. (2) Negative cognition and beliefs. Four studies (14, 24, 26, 31) suggested that negative perceptions of health, aging, or social relationships, as well as uncertainty about the future, may indicate a higher likelihood of loneliness.

### 3.5 Evaluation tools

The 21 included studies used five types of loneliness assessment tools, including multi-item standardized scales, short-form scales, and single-item self-reported questions. Overall, the UCLA Loneliness Scale and its short-form versions were the most commonly used instruments across the included studies, supplemented by the De Jong Gierveld Loneliness Scale and single-item subjective loneliness assessments. These tools differed in the number of items, measurement dimensions, and applicable settings.

#### 3.5.1 The UCLA Loneliness Scale and its abbreviated versions

The UCLA Loneliness Scale was the most widely used tool among the included studies, with 16 studies using this scale or one of its short-form versions. The original UCLA Loneliness Scale contains 20 items and primarily assesses the discrepancy between an individual’s expected social relationships and actual social experiences. It focuses on the overall level of subjective loneliness and is suitable for comprehensive assessment in both clinical and community populations. In empirical studies, some researchers have used shorter versions to reduce participant burden, including the 8-item ULS-8 scale and the 3-item short-form UCLA Loneliness Scale. The ULS-8 substantially shortens assessment time while retaining core dimensions of loneliness, making it suitable for studies with large sample sizes or restricted survey conditions. The 3-item short-form UCLA Loneliness Scale rapidly identifies the frequency of loneliness through core items related to lack of companionship, feeling left out, and social isolation, and is commonly used in epidemiological surveys or preliminary screening studies. Overall, the UCLA scale family has demonstrated good reliability and validity across different cultural contexts and is currently one of the most representative measurement tools in studies of loneliness among older adults with chronic diseases.

#### 3.5.2 De Jong Gierveld Loneliness Scale

The De Jong Gierveld Loneliness Scale was used in three studies. This scale contains 11 items and distinguishes between dimensions such as emotional loneliness and social loneliness, thereby emphasizing the structural characteristics of deficiencies in interpersonal relationships. Unlike the UCLA Loneliness Scale, which focuses more on subjective feelings, this scale places greater emphasis on differences in types of loneliness experiences. It is suitable for analyzing loneliness across different social relationship contexts and is commonly used in European studies and in some non-English-speaking countries.

#### 3.5.3 Single-item self-report question

Some studies assessed loneliness using a single question, such as “Do you feel lonely?” This method is simple to administer, easy for participants to understand, and suitable for large-scale surveys or resource-limited settings. Previous studies have shown that single-item loneliness measures have moderate to high correlations with multi-item scales (32), and they have therefore been widely used in early studies and epidemiological surveys. However, compared with standardized scales, single-item measures have relatively limited ability to distinguish the severity and types of loneliness.

## 4 Discussion

### 4.1 Analysis of loneliness among older adults with chronic diseases and multimorbidity

This scoping review included 21 studies. The findings showed that the reported proportion of loneliness among older adults with chronic diseases and multimorbidity ranged from 25.0 to 97.0%, suggesting a potentially substantial burden of loneliness in this population. However, this range was wide and should not be simply interpreted as a single epidemiological prevalence estimate. Previous research has indicated that measurement instruments are significant moderators of loneliness prevalence estimates, with estimates derived from single-item questions and the short-form UCLA Loneliness Scale being lower than those derived from the full UCLA Loneliness Scale (33). Loneliness estimates may also be substantially influenced by assessment tools, study settings, such as communities, hospitals, and long-term care institutions, cultural contexts, and population characteristics. Studies using the full UCLA Loneliness Scale tended to report higher detection rates, whereas studies using the 3-item short-form UCLA Loneliness Scale or single-item questions reported relatively lower estimates. This may be because multi-item scales are more likely to capture mild, moderate, and less overt experiences of loneliness, whereas single-item questions may be more strongly influenced by participants’ interpretation, perceived stigma, and willingness to disclose loneliness. Overall, later life itself is associated with a considerable burden of loneliness. This review further suggests that when the vulnerabilities associated with older age overlap with those related to chronic diseases and multimorbidity, loneliness may become more prominent. This may be particularly evident among individuals with multimorbidity, functional limitations, poor self-rated health, insufficient social support, and coexisting psychological distress such as depression or anxiety, in whom loneliness may present as a persistent and interrelated health issue.

### 4.2 Analysis of factors associated with loneliness among older adults with chronic diseases and multimorbidity

#### 4.2.1 Social-demographic factors

1. Marital status was one of the relatively consistent sociodemographic factors associated with loneliness across the included studies. Several studies (12–15) suggested that older adults with chronic diseases and multimorbidity who were widowed, divorced, unmarried, living alone, or living in empty-nest households were more likely to report higher levels of loneliness. One possible explanation is that spouses and co-residing family members often provide emotional support, daily care, and assistance with disease management. When these sources of support are absent, physical limitations and treatment-related stress associated with chronic diseases may be more likely to translate into subjective experiences of loneliness.
2. Educational level was associated with loneliness in most included studies, with lower educational attainment being related to higher levels of loneliness. Older adults with lower educational attainment often have limited health literacy and digital literacy, less understanding of disease- and health-related information, reduced access to healthcare and community resources, and fewer opportunities for social participation. As a result, they may be more likely to experience a sense of helplessness during self-management (15, 22, 24, 27), placing them in a more vulnerable position for loneliness during long-term disease management. However, educational level itself does not necessarily directly lead to loneliness; its role may be jointly influenced by economic status, occupational experience, urban–rural differences, health status, and social support.
3. Economic status was also a commonly reported sociodemographic factor associated with loneliness. Several studies (13, 14, 20, 26, 30) suggested that older adults with chronic diseases and multimorbidity who had lower income or greater financial burden reported higher levels of loneliness. Patients with chronic diseases and multimorbidity often require long-term medication, regular follow-up visits, and rehabilitation. Insufficient income may limit expenditures related to healthcare visits, rehabilitation, and cultural or recreational activities, thereby potentially intensifying disease-related social withdrawal and psychological stress (14, 23). Economic status may impose more direct constraints on the daily lives of older patients and may be associated with loneliness by affecting healthcare accessibility, participation in community activities, and the use of social support. However, because income level, health insurance coverage, and financial burden were measured differently across studies, and because some studies did not fully adjust for disease severity, family support, and functional status, the relationship between economic status and loneliness should still be interpreted with caution (18, 23, 26, 30).
4. The relationship between age and loneliness was not fully consistent across the included studies. Some studies (11, 14, 16, 24, 31) suggested that increasing age was associated with higher levels of loneliness. This may be related to the fact that very old adults are more likely to experience loss of a spouse, reduction in peer networks, accumulation of chronic diseases, functional decline, and restricted social activities. However, several studies did not report age as a major associated factor (17, 19, 25, 29), suggesting that age may not serve as a stable independent correlate of loneliness in all older adults with chronic diseases and multimorbidity. International evidence further indicates (34) that the relationship between loneliness and age may not be a simple unidirectional linear relationship, but may be jointly shaped by sex, cultural context, marital status, living arrangement, functional status, and social support.
5. The direction of the association between sex and loneliness was also inconsistent. Cross-checking by study region, care setting, and measurement tool showed that studies reporting higher levels of loneliness among women were conducted in Hong Kong, China, the United States, and Türkiye. These studies included patients with chronic diseases and multimorbidity in primary care, community-dwelling older adults with diabetes, and home-dwelling older adults, and used the De Jong Gierveld Loneliness Scale or the 3-item short-form UCLA Loneliness Scale (22, 25, 30). In contrast, studies reporting higher levels of loneliness among men mainly involved nursing home residents or retired older adults in China and used the UCLA Loneliness Scale (11). These findings suggest that the association between sex and loneliness did not show a stable or consistent pattern across geographical regions or measurement tools. Previous cross-national research (34) and a meta-analysis on loneliness among older adults in China (35) also suggested that levels of loneliness may be influenced by cultural context, family structure, intergenerational support, long-term care services, and community resources, and that age, sex, and cultural factors may interact with each other. In addition, older patients in hospital, rehabilitation, or disease-specific clinical settings usually have more pronounced disease burden, treatment dependence, and functional limitations, whereas community, home-based, or rural samples may be more strongly influenced by family support, neighborhood relationships, and community resources. Therefore, loneliness proportions across different settings should not be directly compared, but should be interpreted in relation to disease type, number of chronic diseases, functional status, and level of social support (21, 22, 36). Meanwhile, differences in loneliness assessment tools, including the number of items, measurement dimensions, cut-off values, and applicable settings, may also contribute to inconsistent loneliness estimates (33, 37). Therefore, among older adults with chronic diseases and multimorbidity, sex should not be interpreted in isolation as a stable factor associated with loneliness; rather, it should be understood in relation to marital status, living arrangement, social support, chronic disease burden, functional status, and depression or anxiety.

Overall, sociodemographic factors are better regarded as indicators for early identification and population stratification, rather than as single or direct intervention targets. In clinical and community practice, information on marital status, living arrangement, educational level, and economic income can be used to prioritize the identification of vulnerable groups, followed by further assessment of social support, functional status, and mental health.

#### 4.2.2 Lifestyle and social support factors

(1) Insufficient social support was a relatively consistent factor associated with loneliness among older adults with chronic diseases and multimorbidity. Several studies reported associations between loneliness and family support, contact with friends, neighborhood relationships, community support, and levels of social participation (21, 24, 29). According to Social Support Theory, social support includes not only emotional companionship but also resources such as disease-related care, access to health information, transportation assistance, daily living support, and maintenance of social roles (38, 39). For older adults with chronic diseases and multimorbidity, long-term medication use, recurrent symptoms, and functional decline may weaken their ability to participate socially. Adequate social support may help alleviate, to some extent, the psychological stress associated with disease burden and may enhance patients’ ability to cope with illness and maintain social roles. (2) Lifestyle was also an important factor closely associated with loneliness. Some studies suggested that regular physical activity, community activities, cultural and recreational activities, and peer interaction were associated with lower levels of loneliness (18, 25, 29, 31). These activities may be associated with loneliness by increasing opportunities for social contact, maintaining functional status, enhancing a sense of belonging, and improving emotional well-being. According to the Cognitive Discrepancy Theory of Loneliness (40), loneliness arises from the discrepancy between an individual’s desired social relationships and actual social relationships. Therefore, simply increasing the frequency of contact may not necessarily reduce loneliness; the key issue is whether social connections can meet patients’ needs for emotional support, being understood, and a sense of belonging. Therefore, future interventions should not be limited to “increasing companionship” alone, but should aim to establish sustainable social connection support systems within chronic disease management settings.

#### 4.2.3 Health status and disease-related factors

Multimorbidity, functional impairment, poor self-rated health, sleep problems, hearing impairment, nutritional status, and polypharmacy have been reported to be associated with levels of loneliness among older adults with chronic diseases and multimorbidity (20, 21, 23, 26, 27, 30, 31). Among these factors, multimorbidity, functional impairment, and poor self-rated health were reported more frequently across the included studies, with relatively consistent evidence (13, 20, 21, 27, 30). In contrast, sleep problems, hearing impairment, nutritional status, and polypharmacy were reported mainly in a limited number of studies or in specific populations, and the evidence for these factors remains relatively limited (19, 26, 30). (1) Chronic diseases and multimorbidity may be associated with loneliness through multiple pathways. National survey data have shown that chronic diseases and multimorbidity are associated not only with increased symptom burden, healthcare expenditure, and care needs, but also with changes in patients’ mobility, social roles, and daily living arrangements (41). For older patients, long-term medication use, frequent follow-up visits, recurrent symptoms, and dependence on others for care may reduce opportunities for going out, social interaction, and participation in community activities, thereby being associated with higher levels of loneliness (42). The Stress Process Model suggests that chronic diseases and multimorbidity can be regarded as persistent stressors, whereas functional decline, financial burden, and reduced social participation may constitute secondary stressors; loneliness may represent a subjective experience arising from the interaction between these stressors and individual psychosocial resources (43). Therefore, the number of chronic diseases or disease burden itself should not be interpreted in isolation as a direct cause of loneliness, but should be understood together with functional status, psychological symptoms, social support, and living environment. (2) Functional impairment and loneliness may have a bidirectional relationship. Longitudinal evidence suggests that loneliness, social isolation, and subsequent functional decline may influence one another (44). Loneliness may be an outcome of chronic diseases, multimorbidity, and functional impairment, but it may also be associated with further functional decline through reduced activity, lower confidence in self-management, and weakened health behaviors. Therefore, among older adults with chronic diseases and multimorbidity, assessment of functional status is important not only for disease management but also as a key indicator for identifying individuals who may be vulnerable to loneliness. Overall, interventions for older adults with chronic diseases and multimorbidity should avoid focusing solely on a single disease. Primary healthcare providers and nursing professionals may assess the number of coexisting chronic diseases, functional health status, hearing and sleep status, nutritional status, and medication burden during chronic disease follow-up. These assessments may be combined with exercise rehabilitation, nutritional support, symptom management, and health education to reduce the continued restriction of social participation associated with disease burden.

#### 4.2.4 Psychological and cognitive factors

Psychological and cognitive factors, such as depression, anxiety, negative illness perceptions, and low self-efficacy, were frequently reported to be associated with levels of loneliness in the included studies (14, 17, 21, 24–26). Patients with chronic diseases and multimorbidity are often exposed to recurrent symptoms, treatment uncertainty, and the stress of functional decline over a prolonged period, which may contribute to negative illness perceptions and feelings of helplessness. When patients perceive their illness as difficult to control, anticipate a decline in future quality of life, or view themselves as a burden to their families, they may be less likely to engage in active social interaction and disease self-management, which may be associated with higher levels of loneliness (36). The study by Lee et al. (45) further suggested that loneliness and depression may have a mutually predictive relationship: depressive symptoms may contribute to loneliness through reduced interest, social avoidance, and decreased activity, whereas loneliness may increase vulnerability to depression through sleep disturbance, deterioration in health behaviors, and heightened stress responses. From the perspective of potential biological and behavioral pathways, loneliness and social isolation may be associated with activation of stress responses, elevated inflammatory levels, sleep disturbance, and deterioration in health behaviors, and these factors may jointly contribute to increased cardiovascular and metabolic risk (46, 47). Therefore, clinical practice should consider integrating mental health management with loneliness risk identification, routinely combining loneliness screening with assessment of depression and anxiety, and implementing stratified interventions for vulnerable groups. For individuals with mild to moderate loneliness, health education, peer support, activity promotion, and family communication may be emphasized. For those with evident depression or anxiety, timely referral to psychological or mental health services should be considered, together with follow-up management within a collaborative care model (48). Future research may integrate disease-related, functional, social relationship, and psychological dimensions to explore the effects of integrated interventions on loneliness.

### 4.3 Cautionary statement on practice implications

The recommendations proposed in this review regarding functional maintenance, psychological support, social participation, and integrated management are mainly based on theoretical synthesis of repeatedly reported associated factors in the included observational studies, rather than on a direct evaluation of intervention effects in this review. Therefore, these recommendations should be understood as potential practice implications and directions for future intervention research, rather than as interventions with established effectiveness. Future well-designed longitudinal studies and randomized controlled trials are still needed to further clarify the causal relationships among factors associated with loneliness and to verify the actual effects of functional improvement, psychological support, social participation promotion, and multicomponent integrated interventions among older adults with chronic diseases and multimorbidity.

### 4.4 Limitations

First, most of the included studies were cross-sectional, making it difficult to determine the causal directions among chronic diseases and multimorbidity, functional impairment, social support, psychological problems, and loneliness (49). Therefore, the factors summarized in this review should be understood as factors associated with loneliness or consistently reported correlates, rather than as established risk factors. Second, the included studies varied substantially in study populations, definitions of chronic diseases and multimorbidity, sample sources, assessment tools, and criteria for identifying loneliness, which limited direct comparison across studies. Third, some studies measured social support, functional status, and psychological factors in a relatively limited manner, which made it difficult to fully explain the mediating or moderating roles among these factors. In addition, this review included only peer-reviewed literature published in Chinese or English and did not include grey literature, conference abstracts, or unpublished studies. Because scoping reviews emphasize broad mapping of evidence in a given field, excluding grey literature may have led to the omission of some practice-based or unpublished evidence, thereby affecting the completeness of the evidence map. Finally, because this review included only Chinese- and English-language literature, language bias may also exist. Future research may combine longitudinal studies, intervention studies, qualitative research, and mixed-methods studies to further explain the mechanisms underlying loneliness and intervention needs among older adults with chronic diseases and multimorbidity.

## 5 Conclusion

Older adults with chronic diseases and multimorbidity experience relatively high levels of loneliness, which are associated with multiple factors, including sociodemographic characteristics, disease and functional status, lifestyle and social support, and psychological and cognitive factors. Among these factors, insufficient social support, multimorbidity, functional impairment, depression and anxiety, living alone, and widowhood were repeatedly reported across multiple studies. Given that the current evidence mainly comes from observational studies and that this review did not directly evaluate the effectiveness of interventions, these factors are more appropriately considered as reference indicators for early identification of loneliness, population stratification, and the design of future intervention studies. Future clinical nursing and primary public health practice may consider incorporating loneliness screening into routine assessments of older adults with chronic diseases and multimorbidity, and exploring integrated management strategies focusing on social support, functional maintenance, mental health, and community participation. Future research should conduct high-quality longitudinal and intervention studies to clarify causal relationships and verify the effectiveness of multicomponent interventions, thereby providing evidence for the development of more precise and scalable strategies to address loneliness among older adults with chronic diseases and multimorbidity.
