# Supplementary material for: Loneliness among older adults with chronic diseases and multimorbidity: a scoping review of prevalence and associated factors
Source: Front Public Health. 2026 Jul 31;14:1846531. doi: 10.3389/fpubh.2026.1846531 (PMC13472916; doi:10.3389/fpubh.2026.1846531)
Supplement: Supplementary file 1 [file Table_1.docx]

For PubMed ：

("Aged"[MeSH Terms] OR "aged patient"[Title/Abstract] OR "aged people"[Title/Abstract] OR "aged person"[Title/Abstract] OR "aged subject"[Title/Abstract] OR "old"[Title/Abstract] OR "older"[Title/Abstract] OR "older adults"[Title/Abstract] OR "old population"[Title/Abstract] OR "old people"[Title/Abstract] OR "elder"[Title/Abstract] OR "elderly"[Title/Abstract] OR "elderly people"[Title/Abstract] OR "elderly patient"[Title/Abstract] OR "elderly person"[Title/Abstract] OR "elderly subject"[Title/Abstract] OR "elder adults"[Title/Abstract] OR "elder people"[Title/Abstract] OR "senior citizen"[Title/Abstract] OR "senium"[Title/Abstract] OR "senior"[Title/Abstract] OR "geriatric"[Title/Abstract])
AND ("Chronic Disease"[MeSH Terms] OR "disease, chronic"[Title/Abstract]
OR "chronic illness*"[Title/Abstract] OR "illness, chronic"[Title/Abstract] OR "chronically ill"[Title/Abstract] OR "chronic condition*"[Title/Abstract] OR "condition, chronic"[Title/Abstract] OR "noncommunicable disease"[Title/Abstract])
AND ("Loneliness"[MeSH Terms] OR "lonely"[Title/Abstract] OR "homesickness"[Title/Abstract] OR "social loneliness"[Title/Abstract])
AND (factor*[Title/Abstract] OR "risk factor*"[Title/Abstract] OR "factor, risk"[Title/Abstract] OR "population* at risk"[Title/Abstract] OR "risk score*"[Title/Abstract] OR "score, risk"[Title/Abstract] OR "risk factor score*"[Title/Abstract] OR "social risk factor*"[Title/Abstract] OR "hazard factor*"[Title/Abstract] OR "influence factor*"[Title/Abstract] OR "correlative factor"[Title/Abstract] OR "associated factor*"[Title/Abstract] OR "adverse effect*"[Title/Abstract] OR "adverse reaction*"[Title/Abstract] OR "cause*"[Title/Abstract] OR "predictor*"[Title/Abstract] OR "clinical predictor*"[Title/Abstract] OR "reason"[Title/Abstract] OR "health correlates"[Title/Abstract] OR "root cause analysis"[Title/Abstract] OR "root cause analyses"[Title/Abstract])

For Web Of Science：

#1 TS=("aged" OR "aged patient" OR "aged people" OR "aged person" OR "aged subject" OR "old" OR "older" OR "older adults" OR "old population"
OR "old people" OR "elder" OR "elderly" OR "elderly people" OR "elderly patient" OR "elderly person" OR "elderly subject" OR "elder adults" OR "elder people" OR "senior citizen" OR "senium" OR "senior" OR "geriatric")

#2 TS=("chronic disease" OR "disease, chronic" OR "chronic illness*" OR "illness, chronic" OR "chronically ill" OR "chronic condition*" OR "condition, chronic" OR "noncommunicable disease")

#3 TS=("loneliness" OR "lonely" OR "homesickness" OR "social loneliness")

#4 TS=(factor* OR "risk factor*" OR "factor, risk" OR "population* at risk"
OR "risk score*" OR "score, risk" OR "risk factor score*" OR "social risk factor*" OR "hazard factor*" OR "influence factor*" OR "correlative factor"
OR "associated factor*" OR "adverse effect*" OR "adverse reaction*"
OR "cause*" OR "predictor*" OR "clinical predictor*" OR "reason"
OR "health correlates" OR "root cause analysis" OR "root cause analyses")

#1 AND #2 AND #3 AND #4

For The Cochrane Library ：

#1 [mh "Aged"] OR "aged patient",ab,kw OR "aged people",ab,kw OR "aged person",ab,kw OR "aged subject",ab,kw OR "old",ab,kw OR "older",ab,kw OR "older adults",ab,kw OR "old population",ab,kw OR "old people",ab,kw OR "elder",ab,kw OR "elderly",ab,kw OR "elderly people",ab,kw OR "elderly patient",ab,kw OR "elderly person",ab,kw OR "elderly subject",ab,kw OR "elder adults",ab,kw OR "elder people",ab,kw OR "senior citizen",ab,kw OR "senium",ab,kw OR "senior",ab,kw OR "geriatric",ab,kw

#2 [mh "Chronic Disease"] OR "disease, chronic",ab,kw OR "chronic illness",ab,kw OR "illness, chronic",ab,kw OR "chronically ill",ab,kw OR "chronic condition",ab,kw OR "condition, chronic",ab,kw OR "noncommunicable disease",ab,kw

#3 [mh "Loneliness"] OR "lonely",ab,kw OR "homesickness",ab,kw OR "social loneliness",ab,kw

#4 [mh "Risk Factors"] OR factor,ab,kw OR "risk factor",ab,kw OR "factor, risk",ab,kw OR "population at risk",ab,kw OR "risk score",ab,kw OR "score, risk",ab,kw OR "risk factor score",ab,kw OR "social risk factor",ab,kw OR "hazard factor",ab,kw OR "influence factor",ab,kw OR "correlative factor",ab,kw OR "associated factor",ab,kw OR "adverse effect",ab,kw OR "adverse reaction",ab,kw OR "cause",ab,kw OR "predictor",ab,kw OR "clinical predictor",ab,kw OR "reason",ab,kw OR "health correlates",ab,kw OR "root cause analysis",ab,kw OR "root cause analyses",ab,kw

#1 AND #2 AND #3 AND #4

For Embase ：

#1 aged'/exp OR aged OR 'aged patient':ab,ti OR 'aged people':ab,ti OR 'aged person':ab,ti OR 'aged subject':ab,ti OR old:ab,ti OR older:ab,ti OR 'the olds':ab,ti OR 'older adults':ab,ti OR 'old population':ab,ti OR 'old people':ab,ti OR elder:ab,ti OR elderly:ab,ti OR 'elderly people':ab,ti OR 'elderly patient':ab,ti OR 'elderly person':ab,ti OR 'elderly subject':ab,ti OR 'elder adults':ab,ti OR 'elder people':ab,ti OR 'senior citizen':ab,ti OR senium:ab,ti OR senior:ab,ti OR geriatric:ab,ti

#2 'chronic disease'/exp OR 'disease, chronic':ab,ti OR 'chronic illness*':ab,ti OR 'illness, chronic':ab,ti OR 'chronically ill':ab,ti OR 'chronic condition*':ab,ti OR 'condition, chronic':ab,ti OR 'noncommunicable disease':ab,ti

#3 'loneliness'/exp OR loneliness OR lonely:ab,ti OR homesickness:ab,ti OR 'emotion loneliness':ab,ti OR 'social loneliness':ab,ti

#4 factor* OR 'risk factor*':ab,ti OR factor，risk:ab,ti OR 'population* at risk':ab,ti OR 'risk score*':ab,ti OR score，risk:ab,ti OR 'risk factor score*':ab,ti OR 'social risk factor*':ab,ti OR 'hazard factor*':ab,ti OR 'influence factor*':ab,ti OR 'correlative factor':ab,ti OR 'nonadherence related factor':ab,ti OR 'associated factor*':ab,ti OR 'adverse effect*':ab,ti OR 'adverse reaction*':ab,ti OR cause*:ab,ti OR predictor*:ab,ti OR 'clinical predictor*':ab,ti OR reason:ab,ti OR 'health correlates':ab,ti OR 'root cause analysis':ab,ti OR 'root cause analyses':ab,ti

#1 AND #2 AND #3 AND #4

For CINAHL ：

(MH "Aged+" OR TI ("aged patient" OR "aged people" OR "aged person" OR "aged subject" OR "old" OR "older" OR "older adults" OR "old population" OR "old people" OR "elder" OR "elderly" OR "elderly people" OR "elderly patient" OR "elderly person" OR "elderly subject" OR "elder adults" OR "elder people" OR "senior citizen" OR "senium" OR "senior" OR "geriatric") OR AB ("aged patient" OR "aged people" OR "aged person" OR "aged subject" OR "old" OR "older" OR "older adults" OR "old population" OR "old people" OR "elder" OR "elderly" OR "elderly people" OR "elderly patient" OR "elderly person" OR "elderly subject" OR "elder adults" OR "elder people" OR "senior citizen" OR "senium" OR "senior" OR "geriatric"))
AND (MH "Chronic Disease+" OR TI ("disease, chronic" OR "chronic illness*" OR "illness, chronic" OR "chronically ill" OR "chronic condition*" OR "condition, chronic" OR "noncommunicable disease")
OR AB ("disease, chronic" OR "chronic illness*" OR "illness, chronic" OR "chronically ill" OR "chronic condition*" OR "condition, chronic" OR "noncommunicable disease"))
AND (MH "Loneliness+" OR TI ("lonely" OR "homesickness" OR "social loneliness") OR AB ("lonely" OR "homesickness" OR "social loneliness"))
AND (MH "Risk Factors+" OR TI (factor* OR "risk factor*" OR "factor, risk" OR "population* at risk" OR "risk score*" OR "score, risk" OR "risk factor score*" OR "social risk factor*" OR "hazard factor*" OR "influence factor*" OR "correlative factor" OR "associated factor*" OR "adverse effect*" OR "adverse reaction*" OR "cause*" OR "predictor*" OR "clinical predictor*" OR "reason" OR "health correlates" OR "root cause analysis" OR "root cause analyses"))

中国知网检索式：

SU=（老年人 + 老人 + 老年 + 老年病人 + 老年患者 + 老年居民 + 老年人口 + 老年人群 + 高龄 + 衰老）AND SU=（慢性病 + 慢性疾病 + 慢病 + 长期患病者 + 非传染性疾病 + 慢性非传染性疾病 + 生活方式病 + 慢性病共病 + 多种慢性病共存状况 + 多种慢性疾病 ）AND SU=（孤独感 + 孤独 + 孤独症 + 思乡病）AND SU=（影响因素分析 + 影响因素 + 危险因素 + 相关因素 + 预测因素 + 风险因素 + 保护因素 + 因素 + 病因）

万方数据库检索式：

(主题:(老年人 OR 老人 OR 老年 OR 老年病人 OR 老年患者 OR 老年居民 OR 老年人口 OR 老年人群 OR 高龄 OR 衰老) and 主题:(慢性病 OR 慢性疾病 OR 慢病 OR 长期患病者 OR 非传染性疾病 OR 慢性非传染性疾病)and 主题:(孤独感 OR 孤独 OR 孤独症 OR 思乡病) and 主题:(影响因素分析 OR 影响因素 OR 危险因素 OR 相关因素 OR 预测因素 OR 风险因素 OR 保护因素 OR 因素 OR 病因))

维普数据库检索式：

M=(老年人 OR 老人 OR 老年 OR 老年病人 OR 老年患者 OR 老年居民 OR 老年人口 OR 老年人群 OR 高龄 OR 衰老) AND M=(慢性病 OR 慢性疾病 OR 慢病 OR 长期患病者 OR 非传染性疾病 OR 慢性非传染性疾病 OR 生活方式病 OR 慢性病共病 OR 多种慢性病共存状况 OR 多种慢性疾病) AND M=(孤独感 OR 孤独 OR 孤独症 OR 思乡病) AND M=(影响因素分析 OR 影响因素 OR 危险因素 OR 相关因素 OR 预测因素 OR 风险因素 OR 保护因素 OR 因素 OR 病因)

中国生物医学文献数据库检索式：

(( "老年人"[常用字段:智能] OR "老人"[常用字段:智能] OR "老年"[常用字段:智能] OR "老年病人"[常用字段:智能] OR "老年患者"[常用字段:智能] OR "老年居民"[常用字段:智能] OR "老年人口"[常用字段:智能] OR "老年人群"[常用字段:智能] OR "高龄"[常用字段:智能] OR "衰老"[常用字段:智能]) AND( "慢性病"[常用字段:智能] OR "慢性疾病"[常用字段:智能] OR "慢病"[常用字段:智能] OR "长期患病者"[常用字段:智能] OR "非传染性疾病"[常用字段:智能] OR "慢性非传染性疾病"[常用字段:智能] OR "生活方式病"[常用字段:智能]) AND( "孤独感"[常用字段:智能] OR "孤独"[常用字段:智能] OR "孤独症"[常用字段:智能] OR "思乡病"[常用字段:智能]) AND( "影响因素分析"[常用字段:智能] OR "影响因素"[常用字段:智能] OR "危险因素"[常用字段:智能] OR "相关因素"[常用字段:智能] OR "预测因素"[常用字段:智能] OR "风险因素"[常用字段:智能] OR "保护因素"[常用字段:智能] OR "因素"[常用字段:智能] OR "病因"[常用字段:智能]))
