# Supplementary material for: Loneliness among older adults with chronic diseases and multimorbidity: a scoping review of prevalence and associated factors
Source: Front Public Health. 2026 Jul 31;14:1846531. doi: 10.3389/fpubh.2026.1846531 (PMC13472916; doi:10.3389/fpubh.2026.1846531)
Supplement: Supplementary file 2 [file Table_2.docx]

A basic methodological quality appraisal was conducted using the appropriate Joanna Briggs Institute critical appraisal tools according to study design. Two reviewers independently appraised each included study. Each item was rated as “yes,” “no,” “unclear,” or “not applicable.”

JBI CRITICAL APPRAISAL CHECKLIST FOR ANALYTICAL CROSS SECTIONAL STUDIES 横断面研究

| Huang Hairong et al.,2017 | | | | |
| --- | --- | --- | --- | --- |
|  | Reviewer  1 | Reviewer 2 | Reviewer 3 | Consensus decisione |
| Were the criteria for inclusion in the sample clearly defined? | Y | Y |  | Y |
| Were the study subjects and the setting described in detail? | Y | Y |  | Y |
| Was the exposure measured in a valid and reliable way? | Y | U | Y | Y |
| Were objective, standard criteria used for measurement of the condition? | U | U |  | U |
| Were confounding factors identified? | Y | Y |  | Y |
| Were strategies to deal with confounding factors stated? | U | Y | Y | Y |
| Were the outcomes measured in a valid and reliable way? | Y | Y |  | Y |
| Was appropriate statistical analysis used? | Y | Y |  | Y |

| Zou Jiayu et al., 2021 | | | | |
| --- | --- | --- | --- | --- |
|  | Reviewer  1 | Reviewer 2 | Reviewer 3 | Consensus decisione |
| Were the criteria for inclusion in the sample clearly defined? | U | U |  | U |
| Were the study subjects and the setting described in detail? | Y | Y |  | Y |
| Was the exposure measured in a valid and reliable way? | U | U |  | U |
| Were objective, standard criteria used for measurement of the condition? | Y | Y |  | Y |
| Were confounding factors identified? | Y | Y |  | Y |
| Were strategies to deal with confounding factors stated? | Y | Y |  | Y |
| Were the outcomes measured in a valid and reliable way? | Y | Y |  | Y |
| Was appropriate statistical analysis used? | Y | Y |  | Y |

| Li Fan et al.,2007 | | | | |
| --- | --- | --- | --- | --- |
|  | Reviewer  1 | Reviewer 2 | Reviewer 3 | Consensus decisione |
| Were the criteria for inclusion in the sample clearly defined? | N | N |  | N |
| Were the study subjects and the setting described in detail? | Y | Y |  | Y |
| Was the exposure measured in a valid and reliable way? | Y | Y |  | Y |
| Were objective, standard criteria used for measurement of the condition? | N | N |  | N |
| Were confounding factors identified? | Y | Y |  | Y |
| Were strategies to deal with confounding factors stated? | U | Y | U | U |
| Were the outcomes measured in a valid and reliable way? | Y | Y |  | Y |
| Was appropriate statistical analysis used? | U | U |  | U |

| Zhang Yuting et al.,2024 | | | | |
| --- | --- | --- | --- | --- |
|  | Reviewer  1 | Reviewer 2 | Reviewer 3 | Consensus decisione |
| Were the criteria for inclusion in the sample clearly defined? | Y | Y |  | Y |
| Were the study subjects and the setting described in detail? | Y | Y |  | Y |
| Was the exposure measured in a valid and reliable way? | U | U |  | U |
| Were objective, standard criteria used for measurement of the condition? | Y | Y |  | Y |
| Were confounding factors identified? | Y | Y |  | Y |
| Were strategies to deal with confounding factors stated? | Y | Y |  | Y |
| Were the outcomes measured in a valid and reliable way? | Y | Y |  | Y |
| Was appropriate statistical analysis used? | Y | Y |  | Y |
| Yang Huanhuan et al.,2022 | | | | |
|  | Reviewer  1 | Reviewer 2 | Reviewer 3 | Consensus decisione |
| Were the criteria for inclusion in the sample clearly defined? | Y | Y |  | Y |
| Were the study subjects and the setting described in detail? | Y | Y |  | Y |
| Was the exposure measured in a valid and reliable way? | Y | Y |  | Y |
| Were objective, standard criteria used for measurement of the condition? | Y | Y |  | Y |
| Were confounding factors identified? | Y | Y |  | Y |
| Were strategies to deal with confounding factors stated? | Y | Y |  | Y |
| Were the outcomes measured in a valid and reliable way? | Y | Y |  | Y |
| Was appropriate statistical analysis used? | Y | Y |  | Y |

| Cheng Shuai et al.,2022 | | | | |
| --- | --- | --- | --- | --- |
|  | Reviewer  1 | Reviewer 2 | Reviewer 3 | Consensus decisione |
| Were the criteria for inclusion in the sample clearly defined? | Y | Y |  | Y |
| Were the study subjects and the setting described in detail? | Y | Y |  | Y |
| Was the exposure measured in a valid and reliable way? | Y | Y |  | Y |
| Were objective, standard criteria used for measurement of the condition? | Y | Y |  | Y |
| Were confounding factors identified? | Y | Y |  | Y |
| Were strategies to deal with confounding factors stated? | Y | Y |  | Y |
| Were the outcomes measured in a valid and reliable way? | Y | Y |  | Y |
| Was appropriate statistical analysis used? | Y | Y |  | Y |

| Huai Yijie et al.,2025 | | | | |
| --- | --- | --- | --- | --- |
|  | Reviewer  1 | Reviewer 2 | Reviewer 3 | Consensus decisione |
| Were the criteria for inclusion in the sample clearly defined? | Y | Y |  | Y |
| Were the study subjects and the setting described in detail? | Y | Y |  | Y |
| Was the exposure measured in a valid and reliable way? | Y | Y |  | Y |
| Were objective, standard criteria used for measurement of the condition? | Y | Y |  | Y |
| Were confounding factors identified? | Y | Y |  | Y |
| Were strategies to deal with confounding factors stated? | Y | Y |  | Y |
| Were the outcomes measured in a valid and reliable way? | Y | Y |  | Y |
| Was appropriate statistical analysis used? | Y | Y |  | Y |

| Li Chao et al.,2018 | | | | |
| --- | --- | --- | --- | --- |
|  | Reviewer  1 | Reviewer 2 | Reviewer 3 | Consensus decisione |
| Were the criteria for inclusion in the sample clearly defined? | Y | U | Y | Y |
| Were the study subjects and the setting described in detail? | Y | Y |  | Y |
| Was the exposure measured in a valid and reliable way? | Y | Y |  | Y |
| Were objective, standard criteria used for measurement of the condition? | Y | Y |  | Y |
| Were confounding factors identified? | Y | Y |  | Y |
| Were strategies to deal with confounding factors stated? | Y | Y |  | Y |
| Were the outcomes measured in a valid and reliable way? | Y | Y |  | Y |
| Was appropriate statistical analysis used? | Y | Y |  | Y |

| Qin Yuqian et al.,2023 | | | | |
| --- | --- | --- | --- | --- |
|  | Reviewer  1 | Reviewer 2 | Reviewer 3 | Consensus decisione |
| Were the criteria for inclusion in the sample clearly defined? | Y | Y |  | Y |
| Were the study subjects and the setting described in detail? | Y | Y |  | Y |
| Was the exposure measured in a valid and reliable way? | Y | Y |  | Y |
| Were objective, standard criteria used for measurement of the condition? | Y | Y |  | Y |
| Were confounding factors identified? | Y | Y |  | Y |
| Were strategies to deal with confounding factors stated? | Y | Y |  | Y |
| Were the outcomes measured in a valid and reliable way? | Y | Y |  | Y |
| Was appropriate statistical analysis used? | Y | Y |  | Y |

| Liu Xi et al.,2022 | | | | |
| --- | --- | --- | --- | --- |
|  | Reviewer  1 | Reviewer 2 | Reviewer 3 | Consensus decisione |
| Were the criteria for inclusion in the sample clearly defined? | Y | Y |  | Y |
| Were the study subjects and the setting described in detail? | Y | U | U | U |
| Was the exposure measured in a valid and reliable way? | U | U |  | U |
| Were objective, standard criteria used for measurement of the condition? | Y | Y |  | Y |
| Were confounding factors identified? | Y | Y |  | Y |
| Were strategies to deal with confounding factors stated? | Y | Y |  | Y |
| Were the outcomes measured in a valid and reliable way? | Y | Y |  | Y |
| Was appropriate statistical analysis used? | Y | Y |  | Y |

| Vespa et al.,2023 | | | | |
| --- | --- | --- | --- | --- |
|  | Reviewer  1 | Reviewer 2 | Reviewer 3 | Consensus decisione |
| Were the criteria for inclusion in the sample clearly defined? | Y | Y |  | Y |
| Were the study subjects and the setting described in detail? | Y | Y |  | Y |
| Was the exposure measured in a valid and reliable way? | Y | Y |  | Y |
| Were objective, standard criteria used for measurement of the condition? | Y | Y |  | Y |
| Were confounding factors identified? | Y | Y |  | Y |
| Were strategies to deal with confounding factors stated? | Y | Y |  | Y |
| Were the outcomes measured in a valid and reliable way? | Y | Y |  | Y |
| Was appropriate statistical analysis used? | Y | Y |  | Y |

| Jiao Zhang et al.,2018 | | | | |
| --- | --- | --- | --- | --- |
|  | Reviewer  1 | Reviewer 2 | Reviewer 3 | Consensus decisione |
| Were the criteria for inclusion in the sample clearly defined? | Y | Y |  | Y |
| Were the study subjects and the setting described in detail? | Y | Y |  | Y |
| Was the exposure measured in a valid and reliable way? | U | U |  | U |
| Were objective, standard criteria used for measurement of the condition? | U | U |  | U |
| Were confounding factors identified? | Y | Y |  | Y |
| Were strategies to deal with confounding factors stated? | Y | Y |  | Y |
| Were the outcomes measured in a valid and reliable way? | Y | U | U | U |
| Was appropriate statistical analysis used? | Y | Y |  | Y |

| Sandeep Grover et al.,2019 | | | | |
| --- | --- | --- | --- | --- |
|  | Reviewer  1 | Reviewer 2 | Reviewer 3 | Consensus decisione |
| Were the criteria for inclusion in the sample clearly defined? | Y | Y |  | Y |
| Were the study subjects and the setting described in detail? | Y | Y |  | Y |
| Was the exposure measured in a valid and reliable way? | U | Y | Y | Y |
| Were objective, standard criteria used for measurement of the condition? | U | U |  | U |
| Were confounding factors identified? | Y | Y |  | Y |
| Were strategies to deal with confounding factors stated? | Y | Y |  | Y |
| Were the outcomes measured in a valid and reliable way? | Y | Y |  | Y |
| Was appropriate statistical analysis used? | Y | Y |  | Y |

| Emma Cho et al.,2024 | | | | |
| --- | --- | --- | --- | --- |
|  | Reviewer  1 | Reviewer 2 | Reviewer 3 | Consensus decisione |
| Were the criteria for inclusion in the sample clearly defined? | Y | Y |  | Y |
| Were the study subjects and the setting described in detail? | Y | Y |  | Y |
| Was the exposure measured in a valid and reliable way? | U | Y | U | U |
| Were objective, standard criteria used for measurement of the condition? | U | U |  | U |
| Were confounding factors identified? | Y | Y |  | Y |
| Were strategies to deal with confounding factors stated? | Y | Y |  | Y |
| Were the outcomes measured in a valid and reliable way? | Y | U | Y | Y |
| Was appropriate statistical analysis used? | Y | Y |  | Y |

| Theeke et al.,2013 | | | | |
| --- | --- | --- | --- | --- |
|  | Reviewer  1 | Reviewer 2 | Reviewer 3 | Consensus decisione |
| Were the criteria for inclusion in the sample clearly defined? | Y | Y |  | Y |
| Were the study subjects and the setting described in detail? | Y | Y |  | Y |
| Was the exposure measured in a valid and reliable way? | U | Y | Y | Y |
| Were objective, standard criteria used for measurement of the condition? | Y | Y |  | Y |
| Were confounding factors identified? | Y | Y |  | Y |
| Were strategies to deal with confounding factors stated? | U | U |  | U |
| Were the outcomes measured in a valid and reliable way? | Y | Y |  | Y |
| Was appropriate statistical analysis used? | Y | Y |  | Y |

| Hacihasanoğlu et al.,2012 | | | | |
| --- | --- | --- | --- | --- |
|  | Reviewer  1 | Reviewer 2 | Reviewer 3 | Consensus decisione |
| Were the criteria for inclusion in the sample clearly defined? | Y | Y |  | Y |
| Were the study subjects and the setting described in detail? | Y | Y |  | Y |
| Was the exposure measured in a valid and reliable way? | U | Y | Y | Y |
| Were objective, standard criteria used for measurement of the condition? | U | U |  | U |
| Were confounding factors identified? | Y | Y |  | Y |
| Were strategies to deal with confounding factors stated? | U | U |  | U |
| Were the outcomes measured in a valid and reliable way? | Y | Y |  | Y |
| Was appropriate statistical analysis used? | Y | Y |  | Y |

| Ohta et al.,2024 | | | | |
| --- | --- | --- | --- | --- |
|  | Reviewer  1 | Reviewer 2 | Reviewer 3 | Consensus decisione |
| Were the criteria for inclusion in the sample clearly defined? | Y | Y |  | Y |
| Were the study subjects and the setting described in detail? | Y | Y |  | Y |
| Was the exposure measured in a valid and reliable way? | U | Y | Y | Y |
| Were objective, standard criteria used for measurement of the condition? | U | U |  | U |
| Were confounding factors identified? | Y | Y |  | Y |
| Were strategies to deal with confounding factors stated? | Y | Y |  | Y |
| Were the outcomes measured in a valid and reliable way? | Y | U | Y | Y |
| Was appropriate statistical analysis used? | Y | Y |  | Y |

| Sönmez Sari et al.,2024 | | | | |
| --- | --- | --- | --- | --- |
|  | Reviewer  1 | Reviewer 2 | Reviewer 3 | Consensus decisione |
| Were the criteria for inclusion in the sample clearly defined? | Y | Y |  | Y |
| Were the study subjects and the setting described in detail? | Y | Y |  | Y |
| Was the exposure measured in a valid and reliable way? | U | Y | Y | Y |
| Were objective, standard criteria used for measurement of the condition? | U | U |  | U |
| Were confounding factors identified? | Y | Y |  | Y |
| Were strategies to deal with confounding factors stated? | Y | Y |  | Y |
| Were the outcomes measured in a valid and reliable way? | Y | Y |  | Y |
| Was appropriate statistical analysis used? | Y | Y |  | Y |

JBI Critical Appraisal Checklist for Cohort Studies 前瞻性队列研究

| SYS Wong et al.,2020 | | | | |
| --- | --- | --- | --- | --- |
|  | Reviewer  1 | Reviewer 2 | Reviewer 3 | Consensus decisione |
| Were the two groups similar and recruited from the same population? | Y | Y |  | Y |
| Were the exposures measured similarly to assign people to both exposed and unexposed groups? | Y | N | N | N |
| Was the exposure measured in a valid and reliable way? | Y | Y |  | Y |
| Were confounding factors identified? | Y | Y |  | Y |
| Were strategies to deal with confounding factors stated? | Y | Y |  | Y |
| Were the groups/participants free of the outcome at the start of the study (or at the moment of exposure)? | U | U |  | U |
| Were the outcomes measured in a valid and reliable way? | Y | Y |  | Y |
| Was the follow up time reported and sufficient to be long enough for outcomes to occur? | Y | Y |  | Y |
| Was follow up complete, and if not, were the reasons to loss to follow up described and explored? | U | U |  | U |
| Were strategies to address incomplete follow up utilized? | U | U |  | U |
| Was appropriate statistical analysis used? | Y | Y |  | Y |

| Kandola et al.,2023 | | | | |
| --- | --- | --- | --- | --- |
|  | Reviewer  1 | Reviewer 2 | Reviewer 3 | Consensus decisione |
| Were the two groups similar and recruited from the same population? | Y | Y |  | Y |
| Were the exposures measured similarly to assign people to both exposed and unexposed groups? | Y | Y |  | Y |
| Was the exposure measured in a valid and reliable way? | Y | Y |  | Y |
| Were confounding factors identified? | Y | Y |  | Y |
| Were strategies to deal with confounding factors stated? | Y | Y |  | Y |
| Were the groups/participants free of the outcome at the start of the study (or at the moment of exposure)? | U | U |  | U |
| Were the outcomes measured in a valid and reliable way? | Y | Y |  | Y |
| Was the follow up time reported and sufficient to be long enough for outcomes to occur? | Y | Y |  | Y |
| Was follow up complete, and if not, were the reasons to loss to follow up described and explored? | U | U |  | U |
| Were strategies to address incomplete follow up utilized? | U | U |  | U |
| Was appropriate statistical analysis used? | Y | Y |  | Y |

JBI CRITICAL APPRAISAL CHECKLIST FOR CASE CONTROL STUDIES病例对照研究

| Chunmei Xia et al.,2023 | | | | |
| --- | --- | --- | --- | --- |
|  | Reviewer  1 | Reviewer 2 | Reviewer 3 | Consensus decisione |
| Were the groups comparable other than the presence of disease in cases or the absence of disease in controls? | Y | U | U | U |
| Were cases and controls matched appropriately? | Y | Y |  | Y |
| Were the same criteria used for identification of cases and controls? | Y | Y |  | Y |
| Was exposure measured in a standard, valid and reliable way? | Y | Y |  | Y |
| Was exposure measured in the same way for cases and controls? | Y | Y |  | Y |
| Were confounding factors identified? | Y | Y |  | Y |
| Were strategies to deal with confounding factors stated? | U | Y | U | U |
| Were outcomes assessed in a standard, valid and reliable way for cases and controls? | Y | Y |  | Y |
| Was the exposure period of interest long enough to be meaningful? | N | N |  | N |
| Was appropriate statistical analysis used? | Y | Y |  | Y |
